# Knowledge and attitude towards sexual and reproductive health rights and associated factors among Adet Tana Haik College students, Northwest Ethiopia: a cross-sectional study

**DOI:** 10.1186/s13104-019-4116-4

**Published:** 2019-02-12

**Authors:** Mulat Ayalew, Dabere Nigatu, Getachew Sitotaw, Ayal Debie

**Affiliations:** 1 Yilmana Densa District Health Office, Amhara National Regional State Health Bureau, Adet, Ethiopia; 2 Department of Reproductive Health, School of Public Health, College of Medicine and Health Sciences, Bahir Dar University, Bahir Dar, Ethiopia; 3 Department of Health Informatics, School of Public Health, College of Medicine and Health Sciences, Bahir Dar University, Bahir Dar, Ethiopia; 4 Department of Health Systems and Policy, Institute of Public Health, University of Gondar, P.O. Box: 196, Gondar, Ethiopia

**Keywords:** Knowledge, Attitude, Sexual and reproductive health rights, Students, Ethiopia

## Abstract

**Objective:**

Sexual and reproductive health rights are the rights of all people regardless of their age, sex and other characteristics to make choices about their own sexuality and reproductive issues. However, little is known about the knowledge and attitude towards SRH rights in Ethiopia. Therefore, this study aimed to assess knowledge and attitude towards SRH rights and associated factors among Adet Tana Haik college students in northwest Ethiopia.

**Results:**

Overall, about 59.6% students were knowledgeable and more than half (53.4%) had favorable attitude towards SRH rights. In this study, students attending third-year class (AOR = 2.20;95% CI 1.29, 3.33), discussion with parents (AOR = 3.35;95% CI 1.61, 6.96), respondent’s mothers attended secondary/above school (AOR = 3.01; 95% CI 1.28, 7.13), participation in RH clubs (AOR = 1.72; 95% CI 1.09, 2.70) and favorable attitude towards SRH rights (AOR = 2.41; 95% CI 1.56, 3.74) were significantly associated with knowledge of participants. On the other hand, knowledge of students (AOR = 2.33; 95% CI 1.36, 7.07), participation in RH clubs (AOR = 1.41;95% CI 1.09, 2.20) and discussion with parents (AOR = 2.50; 95% CI 1.15, 5.47) were the predictors for the attitude of students towards SRH rights. Hence, strengthening women’s education, encouraging discussions with parents and participation in RH clubs may improve the knowledge and attitude of students towards SRH rights.

**Electronic supplementary material:**

The online version of this article (10.1186/s13104-019-4116-4) contains supplementary material, which is available to authorized users.

## Introduction

Sexual and Reproductive Health (SRH) is a state of physical, emotional, mental, and social well-being in relation to sexuality; it is not merely the absence of disease, dysfunction, or infirmity in all matters relating to sexuality and the reproductive system. SRH rights therefore include access to SRH care services, such as information relating to sexuality, sexuality education, respect for bodily integrity, choosing own partner, deciding to be sexually active or not, consensual sexual relations and marriage, decision whether or not and when to have children and pursuing a satisfying and pleasurable sexual life [1, 2].

Adolescent SRH is inseparable from all aspects of adolescent health, providing an opportunity for health gain or loss, and is key to poverty alleviation and economic development [3]. As a result, SRH services can be accessed universally through preventing early child marriage, female genital mutilation and sexual violence and exploitation; and accessing family planning services, safe and legal abortions and comprehensive sexuality education for girls and boys [4], but SRH problems are increasing because of the increasing rates of sexual activity, early pregnancies and Sexually Transmitted Infections (STIs) [5, 6]. The needs of SRH services for young people were also not well understood in many parts of the world [7]. Early and unprotected sexual activity and misconceptions about HIV/AIDS are prevalent, particularly among rural dwellers [8]. About 50% of adolescents (15–19) gave birth in most countries as a result of the lack of access, demand and knowledge about SRH services among sexually active adolescent girls [3].

Studies indicated that there is a discrepancy between knowledge, services utilization and access to RH services [9, 10]. Studies in Nigeria revealed that the majority of the adolescents 60.3% in Ikeja and 62.3% in Ikorodu were aware of SRH rights [11]. In Ethiopia, emotional harm caused by childhood sexual abuse appears to undermine normal and healthy psychological development that can enhance victims’ ability to protect their sexual health [12]. Furthermore, childhood marriage is one of the challenges in the country and almost half of married girls gave birth within the age of 15–19 years. Though the legal age for marriage in the country is 18, about 77% of girls gave their first birth before the age of 18 years [13].

In fact, RH needs of the adolescents have been neither researched nor addressed adequately, but some studies in Ethiopia indicated that knowledge of participants about SRH issues varies from 31.6 to 67% [14–18]. Therefore, this study aimed to assess knowledge and attitude towards SRH rights and associated factors among Adet Tana Haik college students, northwest Ethiopia.

## Main text

### Methods

An institution based cross-sectional study was conducted at Adet Tana Haik College students from March to May, 2017. Adet is located 42 km from Bahir Dar (capital city of Amhara National Regional State). The college was opened in 2015 with a total of 732 students. At the moment it was teaching accounting, management, secretarial sciences, and co-operatives in both diploma and degree programs. The source and study population were all Tana Haik college students; students seriously ill during data collection were excluded. Sample size was determined by using single population proportion formula with the assumption of proportion of knowledgeable students about SRH rights at Wolaita Sodo University (54.5%) [14], 95% confidence level, 5% marginal of error and 10% non-response rate. Therefore, the final sample size was 419. Following sample size determination, the students were stratified into first, second and third year students based on their year of study. The sample was then proportionally allocated to each year of study based on the number of students. Finally, the simple random sampling technique was used to select study participants.

Knowledge about SRH rights, the dependent variable, was measured by using 24 questions and each question contains “0 = No” and “1 = Yes” alternatives. As a result, the total score was (0–24) and participants who scored above the mean score was considered as knowledgeable. On the other hand, attitude towards SRH rights was also measured by using 10 questions and each question contains “0 = disagree” and “1 = agree” alternatives. As a result, the total score was (0–10) and participants who scored above the mean score was considered as having favorable attitude towards SRH rights. The reliability of knowledge and attitude measuring items were checked by calculating the Cronbach alpha (α). The Cronbach alpha (α) for knowledge and attitude measuring tools were 0.81 and 0.83, respectively. Accordingly, the content validity was also checked and approved by experts after reviewing each item of the questionnaire for measuring these variables.

Data was collected using a structured self-administered questionnaire which was adopted from reviewing varieties of literatures [11, 14, 15]. The questionnaire first prepared in English was translated to Amharic and translated back to English in order to maintain its consistency. A one-day training was given to the data collectors and supervisors on the objectives and basic techniques of data collection. Pre-test was done on 42 participants at Adet town Technical, Vocational and Educational Training (TVET) college students and any ambiguity and missed points were incorporated in the final version of the questionnaire. Eight diploma graduate clinical nurses were recruited for data collection. The principal investigator and other two B.Sc. degree graduate nurses supervised the data collection process. The data collection was conducted at the same time for reducing information contamination. The collected data was entered and analyzed using Epi Info version 7.1 and SPSS version 20, respectively. Descriptive statistics such as frequencies and percentages were presented using tables and graphs. Binary logistic regression model was used for analysis. Variables with *p* value < 0.2 during bivariable analysis were entered into the multiple logistic regression. Finally, p-value < 0.05 and AOR with 95% CI were used to determine factors significantly associated with the outcome variables during multiple logistic regression.

### Results

#### Socio-demographic characteristics

A total of 416 students participated in the study with a response rate of 99.3%. The mean age of the participants was 21 ± 1.94 years. Nearly 99% of the participants were Orthodox Christians. Female students accounted for 59.6% and two-thirds (68%) of the respondents came from rural areas. More than eighty percent (83.7%) of the participants attended their elementary and secondary education in public schools and 38.2% of students were attending their first-year college education. Their parents’ demographic data showed that 63.7% of their fathers and approximately two-thirds (67.8%) of their mothers were unable to read and write. More than ninety percent (91.1%) of their fathers were farmers, while nearly 85% of their mothers were housewives (Table 1).

**Table 1:** Socio-demographic characteristics of Adet Tana Haik college students Northwest Ethiopia, 2017

Variables	Category	Frequency	Percent (%)
Age in years	15–19	69	16.6
	20–24	319	76.6
	25–29	28	6.8
Sex	Male	168	40.4
	Female	248	59.6
Marital status	Single	350	84.1
	Married	66	15.9
Residence	Urban	133	32.0
	Rural	283	68.0
Living arrangement	Husband/wife	110	26.4
	Parents	40	9.6
	School friends	266	63.9
Department	Accounting	243	58.4
	Management	42	10.1
	Secretary	60	14.4
	Co-operative	71	17.1
Year of study	First	159	38.2
	Second	131	31.5
	Third	126	30.3
Elementary and high school attended	Public	348	83.7
	Private	48	11.5
	Both	20	4.8
Paternal education	Unable to read and write	265	63.7
	Primary school	115	27.6
	Secondary and above	36	8.7
Paternal occupation	Farmer	379	91.1
	Governmental employee	20	4.8
	Merchant	17	4.1
Maternal education	Unable to read and write	282	67.8
	Primary school	95	22.8
	Secondary and above	39	9.4
Maternal occupation	Housewife	355	85.3
	Merchant	31	7.5
	Governmental employee	30	7.2

#### Knowledge and attitude towards SRH Rights

This study revealed that 59.6% of the participants were knowledgeable about SRH rights and more than half (53.4%) of the students had favorable attitude towards SRH rights (Additional file 1: Fig. S1).

#### Factors associated with knowledge of SRH rights

The finding showed that participants who attended their second year (AOR = 1.98;95% CI 1.18, 3.33) and third year classes (AOR = 2.20; 95% CI 1.29, 3.33) were 1.98 and 2.2 times more likely to be knowledgeable, respectively, compared with those who attended their first year of study. Students who had discussed SRH issues with their parents were 3.35 times (AOR = 3.35; 95% CI 1.61, 6.96) more likely to be knowledgeable than students who did not discuss. Students whose mothers had attended primary, secondary and above schools, were 1.91 (AOR = 1.91; 95% CI 1.10, 3.32) and 3.01 (AOR = 3.01; 95% CI 1.28, 7.13) times more likely to be knowledgeable than students whose mothers could not able to read and write. Students who had participated in reproductive health clubs were 1.72 times (AOR = 1.72; 95% CI 1.09, 2.70) more likely to be knowledgeable than students who had not participated. Furthermore, participants who had favorable attitude towards SRH rights were 2.41 times (AOR = 2.41; 95% CI 1.56, 3.74) more likely to be knowledgeable compared with participants who had no favorable attitude (Table 2).

**Table 2:** Factors associated with knowledge of SRH rights among Adet Tana Haik College students, 2017

Variables and categories	Total, n (%)	Knowledge on SRH		COR 95% CI	AOR 95% CI
		Knowledgeable, n (%)	Not knowledgeable, n (%)		
Sex of respondents					
Male (r)	168 (40.38)	108 (25.96)	60 (14.42)	1	1
Female	248 (59.62)	140 (33.65)	108 (25.96)	0.72 (0.48, 1.08)	0.75 (0.48, 1.16)
Marital status					
Single (r)	350 (84.13)	206 (49.52)	144 (34.62)	1	1
Married	66 (15.87)	42 (10.10)	24 (5.77)	1.22 (0.71, 2.11)	1.34 (0.64, 2.83)
SRH discussion with parents					
No (r)	42 (10.10)	14 (3.37)	28 (6.73)	1	1
Yes	374 (89.90)	234 (56.25)	140 (33.65)	3.34 (1.70, 6.57)	3.35 (1.61, 6.96)*
Living arrangement					
Parents (r)	40 (9.62)	24 (5.77)	16 (3.85)	1	1
School friends	266 (63.94)	156 (37.50)	110 (26.68)	0.95 (0.48, 1.86)	1.39 (0.57, 3.43)
Husband/wife	110 (26.44)	68 (16.35)	42 (10.10)	1.08 (0.52, 2.26)	1.51 (0.58, 3.89)
Year of study					
First (r)	159 (38.22)	78 (18.75)	81 (19.47)	1	1
Second	119 (28.61)	74 (17.79)	45 (10.82)	1.71 (1.05, 2.77)	1.98 (1.18, 3.33)*
Third	138 (33.17)	96 (23.08)	42 (10.10	2.37 (1.47, 3.83)	2.20 (1.29, 3.76)*
Maternal education					
Unable to read and write (r)	282 (67.79)	151 (36.30)	131 (31.49)	1	1
Primary school	95 (22.84)	66 (15.87)	29 (6.97)	1.97 (1.20, 3.24)	1.91 (1.10, 3.32)*
Secondary and above	39 (9.38)	31 (7.45)	8 (1.92)	3.36 (1.49, 7.57)	3.01 (1.28, 7.13)*
Participation in RH clubs					
No (r)	222 (53.37)	113 (27.16)	109 (26.20)	1	1
Yes	194 (46.63)	135 (32.45)	59 (14.18)	2.21 (1.47, 3.30)	1.72 (1.09, 2.70)*
Hx of using SRH services					
No (r)	299 (71.88)	169 (40.63)	130 (31.25)	1	1
Yes	117 (28.13)	79 (18.99)	38 (9.13)	1.60 (1.02, 2.51)	1.21 (0.78, 3.65)
Attitude on SRH rights					
Unfavorable (r)	194 (46.63)	99 (23.80)	95 (22.84)	1	1
Favorable	222 (53.37)	149 (35.82)	73 (17.55)	1.96 (1.32, 2.91)	2.41 (1.56, 3.74)*

* Significant at p-value < 0.05, *r* reference category

#### Factors associated with attitude towards SRH rights

In this study, students who were knowledgeable about SRH rights were 2.33 times (AOR = 2.33; 95% CI 1.36, 7.07) more likely to have favorable attitude towards SRH rights compared with students who were not knowledgeable. Respondents who had participated in RH clubs were 1.41 times (AOR = 1.41; 95% CI 1.09, 2.20) more likely to have favorable attitude than participants who did not participate. Students who had discussions with their parents about SRH rights issue were 2.50 times (AOR = 2.50; 95% CI 1.15, 5.47) more likely to have favorable attitude compared with students who had no discussion with their parents (Table 3).

**Table 3:** Factors associated with attitude towards SRH rights among Adet Tana Haik college students, 2017

Variables and categories	Total, n (%)	Attitude towards SRH rights		COR 95% CI	AOR 95% CI
		Favorable, n (%)	Unfavorable, n (%)		
Sex of respondents					
Male (r)	168 (40.38%)	94 (22.60)	74 (17.79)	1	1
Female	248 (59.62)	128 (30.77)	120 (28.85)	0.84 (0.57, 1.25)	0.97 (0.63, 1.49)
Residence					
Urban (r)	133 (31.97)	72 (17.31)	61 (14.66)	1	1
Rural	283 (68.03)	150 (36.06)	133 (31.97)	0.96 (0.63, 1.45)	0.99 (0.62, 1.57)
Maternal education					
Unable to read and write (r)	282 (67.79)	143 (34.38)	139 (33.41)	1	1
Primary school	95 (22.84)	51 (12.26)	44 (10.58)	1.13 (0.75, 1.90)	1.07 (0.66, 1.90)
Secondary and above	39 (9.38)	28 (6.73)	11 (2.64)	2.47 (1.20, 5.24)	1.92 (0.87, 4.23)
Participation in RH clubs					
No (r)	222 (53.37)	103 (24.76)	119 (28.61)	1	1
Yes	194 (46.63)	119 (28.61)	75 (18.47)	1.83 (1.24, 2.71)	1.41 (1.09, 2.20)*
Hx of using SRH services					
No (r)	299 (71.88)	149 (35.82)	150 (36.06)	1	1
Yes	117 (28.13)	73 (17.55)	44 (10.58)	1.67 (1.08, 2.59)	1.33 (0.81, 2.17)
Living arrangement					
Parents (r)	40 (9.62)	22 (5.29)	18 (4.33)	1	1
School friends	266 (63.94)	143 (34.38)	123 (29.57)	0.95 (0.49, 1.86)	0.69 (0.29, 1.67)
Husband/wife	110 (26.44)	57 (13.70)	53 (12.74)	0.88 (0.43, 1.82)	0.65 (0.26, 1.63)
Discussed on SRH rights with parents					
No (r)	42 (10.10)	11 (2.64)	31 (7.45)	1	1
Yes	374 (89.90)	211 (50.72)	163 (39.18)	3.65 (1.78, 7.48)	2.50 (1.15, 5.47)*
Students’ knowledge					
Not knowledgeable (r)	168 (40.40)	73 (17.55)	95 (22.84)	1	1
Knowledgeable	248 (59.60)	149 (35.82)	99 (23.80)	1.96 (1.32, 2.91)	2.33 (1.36, 7.07)*

* Significant at p-value < 0.05, *r* reference category

### Discussion

This study aimed to assess knowledge and attitude towards SRH rights and associated factors among Adet Tana Haik college students. The study found that 59.6% of the participants were knowledgeable about SRH rights. The finding was in line with studies conducted on young people with disability in Ethiopia (64.6%) [17], Wolaita Sodo, Ethiopia (54.5%) [14], Ikorodu (62.3%) and Ikeja (60.3%) [11] and Ikenne (63.2%) [19], Southwest Nigeria. However, the result was lower than those of studies conducted in East Gojjam, Ethiopia (67%) [15] and Ghana (80%) [20] but higher than those of studies in Harar (31.6%) [18] and Shire town (47.1%) [16]. On the other hand, in this study more than half (53.4%) of the participants had favorable attitude towards SRH rights. This finding was lower than the study conducted in Eastern Ethiopia (69.3%) [21]. This variation might be due to the differences in study population, area and period.

The study showed that second and third-year students were 1.98 and 2.2 times more likely to be knowledgeable than their first-year counterparts, respectively. This finding was supported by studies conducted at Wolaita Sodo University [14] and East Gojjam zone [15]. This might be the result of the differences in exposure, information sharing and communication on SRH rights among students in the intermediate and senior classes.

Students who had discussed the SRH rights with their parents were 3.35 times more likely to be knowledgeable compared with their counterparts. This study was also consistent with studies conducted at Wolaita Sodo University and East Gojjam zone, Ethiopia [14, 15].

Students whose mothers had attended primary, secondary and above schools, were 1.91 and 3.01 times more likely to be knowledgeable than whose mothers could not able to read and write. The possible explanation might be students whose mothers attended their primary and above education might give better advice and share the right information about SRH rights during their discussions.

Students participated in RH clubs were 1.72 times more likely to be knowledgeable than their counterparts. This finding was in line with those of studies done at Wolaita Sodo University [14] and Shire town, Ethiopia [16]. This might be because students who had participated in RH clubs might have the opportunity to ask and discuss about SRH rights.

Participants who had favorable attitude towards SRH rights were 2.41 times more likely to be knowledgeable compared with those with unfavorable attitude. This might be so because participants with favorable attitude towards SRH rights might have enhanced their effort to read and discuss with individuals specialized in the area in order to improve their awareness.

On top of that, students who had good knowledge about SRH issues with their parents were 2.33 times more likely to have favorable attitude towards exercising SRH rights. This finding was supported by studies [12, 22, 23]. The possible justification might be due to students who had good knowledge might analyze and understand the risks/benefits and they might have good initiation to exercise their theoretical knowledge into practice.

Our findings also indicated that students who had participated in RH clubs at school and discussed on SRH issues with parents were 1.41 and 2.50 times more likely to have favorable attitude compared with students who had not participated, respectively. This finding was supported by a qualitative finding in Kenya [24]. This might be due to both parents and peers may share their experiences with their peers, parents, and expertise, particularly proper parental guidance on some of the genesis of SRH problems experienced in young people would help the participants to have positive attitude towards SRH services.

Therefore, strengthening women’s education, encouraging discussions with parents and participations in reproductive health clubs at school levels might improve the knowledge and attitude towards SRH rights.

## Limitation of the study

- As a cross sectional attempt with a quantitative design, this work has not shown cause-effect relationships.

## Additional file

**Additional file 1: Fig. S1.** SRH service utilization and source of information among Adet Tana Haik college students northwest Ethiopia, 2017. Nearly one-fifth (18.3%) of the participants had history of sexual intercourse and about 47% had also participated in RH clubs at school level. The source of information for about 29.1% of the respondents were health professionals and nearly 90% of the students had discussed with their parents previously about SRH issues. Furthermore, 28.1% of the students had history of SRH service utilization.

## Abbreviations

ANRHB: Amhara National Regional Health Bureau
ASRH: Adolescent Sexual and Reproductive Health
HIV: Human Immune Virus
STIs: Sexually Transmitted Infections
SRH: Sexual and Reproductive Health
TVET: Technical, Vocational and Educational Training
WHO: World Health Organization
