# Supplementary figures and images for: Knowledge and attitude towards sexual and reproductive health rights and associated factors among Adet Tana Haik College students, Northwest Ethiopia: a cross-sectional study
Source: BMC Res Notes. 2019 Feb 12;12:80. doi: 10.1186/s13104-019-4116-4 (PMC6373003; doi:10.1186/s13104-019-4116-4)

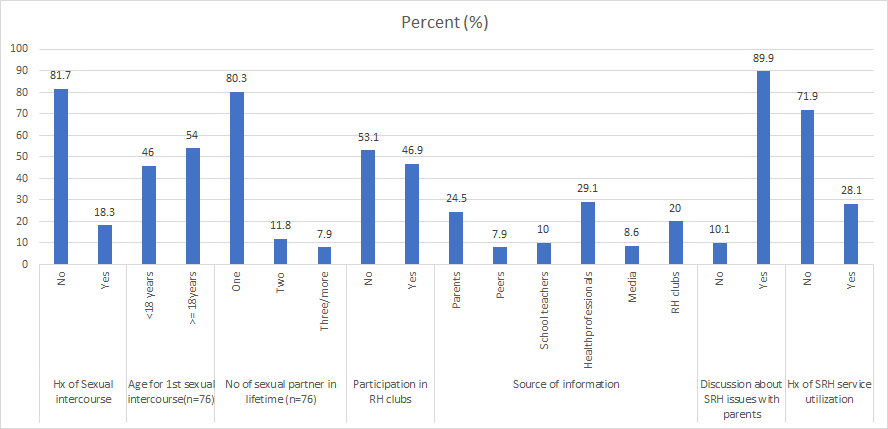

Supplement: Supplementary file 1 — Additional file 1: Fig. S1. SRH service utilization and source of information among Adet Tana Haik college students northwest Ethiopia, 2017. Nearly one-fifth (18.3%) of the participants had history of sexual intercourse and about 47% had also participated in RH clubs at school level. The source of information for about 29.1% of the respondents were health professionals and nearly 90% of the students had discussed with their parents previously about SRH issues. Furthermore, 28.1% of the students had history of SRH service utilization. [file 13104_2019_4116_MOESM1_ESM.tif]
